# Objectively measured physical environmental neighbourhood factors are not associated with accelerometer-determined total sedentary time in adults

**DOI:** 10.1186/s12966-017-0551-6

**Published:** 2017-07-14

**Authors:** Sofie Compernolle, Katrien De Cocker, Joreintje D. Mackenbach, Femke Van Nassau, Jeroen Lakerveld, Greet Cardon, Ilse De Bourdeaudhuij

**Affiliations:** 1 Department of Movement and Sport Sciences, Faculty of Medicine and Health Sciences, Ghent University, Watersportlaan 2, B-9000 Ghent, Belgium; 2 Research Foundation Flanders (FWO), Brussels, Belgium; 3 Department of Epidemiology and Biostatistics, Amsterdam Public Health Research Institute, VU University Medical Center, Amsterdam, The Netherlands; 4 Department of Public and Occupational Health, Amsterdam Public Health Research, VU University Medical Center, Amsterdam, The Netherlands

**Keywords:** Sitting time, Built environment, Adults, Neighborhood

## Abstract

**Background:**

The physical neighbourhood environment may influence adults’ sedentary behaviour. Yet, most studies examining the association between the physical neighbourhood environment and sedentary behaviour rely on self-reported data of either the physical neighbourhood environment and/or sedentary behaviour. The aim of this study was to investigate the associations between objectively measured physical environmental neighbourhood factors and accelerometer-determined total sedentary time in adults.

**Methods:**

In total, 219 Dutch and 128 Belgian adults (mean age ± SD: 55.8 ± 15.4 years) were recruited between March and August 2014 as part of the European SPOTLIGHT project. Physical environmental neighbourhood factors, grouped into eight domains, i.e. walking, cycling, public transport, aesthetics, land use mix, grocery stores, food outlets and recreational facilities, were assessed using the SPOTLIGHT Virtual Audit Tool. Sedentary time was collected using ActiGraph GT3X+ accelerometers. General linear mixed models were conducted to examine associations between physical environmental neighbourhood factors and total sedentary time.

**Results:**

Participants were sedentary, on average, for 542.9 min/day (SD: 84.3), or 9.1 h/day. None of the examined physical environmental neighbourhood factors were significantly related to total sedentary time.

**Conclusions:**

Our findings do not support associations of objectively measured physical environmental neighbourhood factors with adults’ objectively sedentary time in Dutch and Belgian adults. More research on sedentary behaviours in settings such as the home and work setting is needed to examine the influence of more specific physical environmental factors on these context-specific sedentary behaviours.

## Background

The increase in sedentary behaviour, i.e. any waking activity characterized by an energy expenditure of ≤1.5 metabolic equivalents while in a sitting, reclining or lying position [1], is thought to be an important risk factor for obesity and related chronic diseases, including diabetes mellitus type 2 and cardiovascular diseases [2, 3]. The majority of adults spend too much time sedentary during transport, at work, at home and during leisure [4, 5]. In order to reduce sedentary time in adults, more insight into the determinants is needed.

Social ecological models describe the potential role of physical environmental factors for sedentary behaviours [6]. These physical environmental factors can be classified into home and institutional environmental factors (e.g. factors in the work environment) and built and natural environmental factors (e.g. factors of the neighbourhood environment) [7]. Physical environmental neighbourhood factors have been recognised as potential determinants of sedentary behaviour by a panel of experts in the recently developed Systems of Sedentary behaviours (SOS) framework [7]. For example, the presence of benches in the neighbourhood may directly stimulate residents to be sedentary. Indirectly, the lack of sidewalks and cycle paths in a neighbourhood may increase sedentary time as residents may spend less time on active transportation and more time on passive transportation, or the presence of recreational facilities may encourage residents to go outside, and in this way reduce their sedentary time. Identifying physical environmental neighbourhood factors that are associated with sedentary time is important to inform public health practitioners for developing effective population-based interventions. Koohsari et al. recently summarized the evidence on associations between neighbourhood environmental factors and sedentary time in a systematic review [8], concluding that there is mixed evidence, if any, for some modest associations. From the 17 included studies in that review, only two studies investigated the association between objectively measured neighbourhood factors and objectively measured total sedentary time [9, 10]. In the study of Van Dyck et al., examining the association between neighbourhood walkability and sedentary time among 1200 Flemish adults, results showed that residents from high walkable neighbourhoods spent 2.9% more time sedentary than residents from low walkable neighbourhoods [9]. In the study of Kozo et al., conducted among 2199 adults in the USA, no significant association was observed between neighbourhood walkability and total sedentary time [10]. Of note is that both studies examined the role of walkability. Walkability, defined by residential density, street connectivity and land-use mix, is related to the design of a neighbourhood, and can thus be classified as a macro-scale physical environmental factor. Another study, not included in the review of Koohsari et al., has also recently examined the association between objectively measured macro-environmental neighbourhood factors, such as residential density, intersection density and entertainment density, and objectively measured sedentary behaviour [11]. This study concluded that only net residential density and public transit density were associated with sedentary behaviour [11]. All other studies to date, examined micro-scale environmental factors (i.e. regarding the streetscape) and relied on self-reported data of either the environmental attributes or sedentary time, which may be subject to recall and reporting bias [8, 12].

To overcome the limitations of self-reported physical environments and sedentary time, the aim of this study was to examine associations between objectively measured physical environmental neighbourhood factors and accelerometer-determined sedentary time in Dutch and Belgian adults.

## Methods

### Study design and sampling

Data from the present study were derived from the ‘Sustainable prevention of obesity through integrated strategies’ (SPOTLIGHT) study [13]. In this study, a cross-sectional survey was conducted among 6037 adult participants from 60 selected neighbourhoods of five urban regions across Europe: Ghent and suburbs (Belgium), Paris and inner suburbs (France), Budapest and suburbs (Hungary), The Randstad (a conurbation including the cities of Amsterdam, Rotterdam, The Hague, and Utrecht in The Netherlands) and Greater London (UK). Neighbourhoods were defined according to small-scale local administrative boundaries. Sampling of neighbourhoods and recruitment of participants has been described in detail elsewhere [14]. After completing the survey, Dutch and Belgian participants who indicated being interested in future studies, were contacted to participate in an accelerometer study. In total, 255 Dutch and 167 Belgian participants (participation rate = 16%) of 24 different neighbourhoods (i.e. 12 Dutch and 12 Belgian neighbourhoods; mean surface is 1.2 km^2^ and 1.4 km^2^ respectively) agreed to wear an accelerometer for seven consecutive days. In the Netherlands, the accelerometers were sent to the participants’ home addresses including a written instruction on how to wear the device. In Belgium, researchers visited the participants at home to attach the accelerometers and to provide the instructions. In total, informed consents and valid accelerometer data were obtained from 225 Dutch and 149 Belgian participants between March and August 2014. The study was approved by the VU University Medical Centre ethics committee (2012/314) and the Ghent University Hospital ethical committee (EC/2013/518).

### Measures

#### Accelerometer-determined sedentary time

The Actigraph triaxial accelerometer (Model GT3X, Actigraph, LLC, Fort Walton beach, FL) was fixed with an elastic belt around the waist on the right side of the participants. Participants were asked to wear the accelerometer for seven consecutive days during waking hours, except during bathing and other water-based activities. A valid day was defined as a day which contained at least 10 h of accelerometer data, and only adults with at least four valid days were included in the analyses. Each minute of wear-time was classified into sedentary (<100 cpm), light (100–2019 cpm), moderate (2020–5998 cpm) and vigorous intensity activity (>5999 cpm) according to commonly used cut points for adults [15]. Non-wear time was defined as 60 min of consecutive zeroes, allowing for two interruptions of less than 100 counts per minute [15].

#### Objectively measured physical environmental neighbourhood factors

Objectively measured physical environmental factors of the selected neighbourhoods were assessed using the reliable and valid SPOTLIGHT Virtual Audit Tool (S-VAT) [16]. The S-VAT contains 42 items, grouped into eight domains: walking (six items), cycling (eight items), public transport (two items), aesthetics (nine items), land use mix (three items), grocery stores (five items), food outlets (six items) and recreational facility-related items (three items). All items were assessed at street segment level within the 24 selected neighbourhoods in the Netherlands and in Belgium by trained researchers of the SPOTLIGHT project team. Street segment level data were aggregated to the neighbourhood level by taking the percentage of street segments with each feature in the neighbourhood [17]. Items for which no association with sedentary time was expected based on the SOS framework (e.g. liquor stores, type of residential buildings) [7] were excluded from the statistical analyses, as well as items with limited variance, i.e. if more than half of the neighbourhoods yielded the same percentage (e.g. indoor recreational facilities, take away restaurants, abandoned buildings, and railway/underground stations). As a result, 16 physical environmental neighbourhood factors were included in the statistical analyses (see Table 2).

#### Covariates

Covariates considered in the analyses were retrieved from the online survey, and included age, gender, educational level (lower, higher), and household composition (number of people in the household).

### Statistical analysis

Only participants for whom questionnaire data, S-VAT data and valid accelerometer data were available were included in the current analyses (*N* = 347). Descriptive statistics were computed using R software, version 3.1.2. to summarize participant characteristics. General linear mixed models were used to examine the associations between physical environmental neighbourhood factors and sedentary time, as the Kolmogorov-Smirnov test revealed a normal distribution of the dependent variable (sedentary time). A random intercept was added within all models to account for clustering of participants at neighbourhood level. Physical environmental neighbourhood items were multiplied by 100 before entering in the models to facilitate interpretation, so that a 1-unit increase represents a 1% increase in the presence of neighbourhood characteristics.

In a first step, the separate associations between the sixteen selected physical environmental neighbourhood factors and sedentary time were analysed using single regression models. In a second step (model 1), the same physical environmental neighbourhood factors were entered in a multivariable regression model, except the presence of cafés, local food shops, residential gardens, litter, bicycle lanes and public parks. As the variance inflation factors showed that these variables violated the assumption of multicollinearity, these variables were not included in the multivariable regression model. In a final step (model 2), the covariates were added to the model to control for their influence on the potential associations. Statistical significance was set at *p* ≤ 0.05.

## Results

### Participant characteristics

Participant characteristics are presented in Table 1. The mean age of the total sample was 55.8 (SD = 15.4) years, ranging from 19 to 91 years. More than half of the Dutch participants were female (56.7%), whereas more than half of the Belgian participants were male (54.9%). Most participants lived in a two-person household (46.9%), and completed tertiary education. The mean sedentary time was 542.9 min/day, or 9.05 h/day.

**Table 1:** Socio-demographic characteristics, body mass index, and sedentary time of the total sample

	Total (*n* = 347)	Dutch participants (*n* = 219)	Belgian participants (*n* = 128)
Age: years, mean (SD)	55.8 (15.4)	57.6 (15.1)	52.8 (15.5)
Gender: %			
Male	47.5	43.3	54.9
Female	52.5	56.7	45.1
Household composition: %			
One-person household	23.2	27.6	15.7
Two-person household	46.9	44.9	50.4
Three-or more-person household	29.9	27.5	33.9
Educational level: %			
No tertiary education	36.3	40.3	29.3
Tertiary education	63.7	56.7	70.7
Total sedentary time: min/day, mean (SD)	542.9 (84.3)	556.9 (81.8)	518.7 (83.4)

*N* number of participants, *SD* standard deviation

### Physical environmental neighbourhood correlates of accelerometer-determined sedentary time

Table 2 shows the results of the regression analyses. None of the examined physical environmental neighbourhood factors were significantly associated with accelerometer-determined sedentary time, and only one factor showed a trend towards significance with sedentary time, namely the presence of traffic calming devices (*p* = 0.06). This trend was only visible in the single regression analysis.

**Table 2:** Associations between physical environmental neighbourhood factors and total sedentary time (*N* = 347)

Physical environmental neighbourhood factors ^b^	Physical environmental factors separately (*n* = 347)		Model 1: Physical environmental factors combined (*n* = 347)		Model 2: Adjusted for individual factors ^a^ (*n* = 329)	
	b (S.E.) ^c^	p	b (S.E.) ^c^	p	b (S.E.) ^c^	p
Presence of food outlets and grocery stores						
Restaurants	0.36 (1.39)	0.80	1.54 (1.66)	0.38	1.32 (1.74)	0.47
Café/bar	−0.32 (1.59)	0.84	-	-	-	-
Supermarkets	−4.46 (2.71)	0.11	−5.77 (3.48)	0.14	−4.81 (3.75)	0.24
Local food shops	−1.64 (2.35)	0.50	-	-	-	-
Aesthetics						
Green/water area	0.12 (0.20)	0.54	0.09 (0.25)	0.74	−0.02 (0.27)	0.93
Residential gardens	0.02 (0.19)	0.90	-	-	-	-
Litter	0.13 (0.22)	0.55	-	-	-	-
Presence of public transport						
Presence of bus/tram stops	−1.93 (1.50)	0.21	−0.99 (2.00)	0.63	0.14 (2.13)	0.95
Presence of cycling-related items						
Presence of bicycle lanes	0.87 (0.59)	0.16	-	-	-	-
Obstacles on bicycle lanes	−0.97 (0.60)	0.13	−0.80 (0.62)	0.24	−0.75 (0.66)	0.30
Traffic calming devices	0.52 (0.26)	0.06*	0.58 (0.35)	0.13	0.59 (0.37)	0.14
Presence of walking-related items						
Pedestrian crossings	0.43 (0.36)	0.25	−0.10 (0.44)	0.82	0.28 (0.48)	0.59
Presence of side walks	−0.20 (0.19)	0.30	−0.21 (0.22)	0.38	−0.19 (0.24)	0.45
Land-use mix						
Percentage of residential buildings	0.07 (0.74)	0.92	0.25 (0.84)	0.77	0.38 (0.88)	0.68
Presence of physical activity facilities						
Outdoor recreational facilities	2.84 (2.84)	0.33	−3.19 (3.34)	0.37	−3.06 (3.58)	0.42
Public parks	−0.72 (0.98)	0.47	-	-	-	-

*0.05 < *p* < 0.10

^a^Included covariates were age, gender, educational level, and household composition

^b^All physical environmental factors are expressed in the percentage of street segments within a neighbourhood including the physical environmental factor

^c^The b’s represent the increase in total sedentary time (minutes/day) with a one unit increase in the physical environmental factors. For example, if there is an increase of 1% of the street segments within a neighbourhood with outdoor recreational facilities, residents of this neighbourhood will sit 2.84 min more per day

## Discussion

This study examined whether a range of objectively measured physical environmental neighbourhood factors were related to adults’ accelerometer-determined sedentary time. The results showed that none of the examined physical environmental factors were significantly associated with total sedentary time, suggesting that neighbourhood related factors are not influential for the total amount of time people sit per day. This is in contrast with what was expected from Owen’s ecological model of four domains of sedentary behaviour. In this model, the neighbourhood environment is listed as one of the behavioural settings that might influence adults’ sedentary time. Essential to note is that in this model, the importance of four different domains of sedentary behaviour (i.e. occupational, household, transport-related and leisure time sedentary time) has also been emphasized, such that specific sedentary behaviours may happen in a variety of behavioural settings [6]. Only two out of four domains of sedentary behaviour might occur partially in the neighbourhood environment, namely transport-related sedentary behaviour and leisure-time sedentary behaviour (e.g. sitting in a restaurant). These behaviours might thus directly be influenced by the neighbourhood environment. However, these behaviours generally represent only a small part of total sedentary time [18, 19]. This may be one explanation for the lack of associations with total sedentary time in our study. The vast majority of adults’ sedentary time is attributed to occupational sedentary behaviour and recreational television time [18, 19]. Occupational sedentary behaviour generally takes place at the workplace and television time generally takes place at home [6]. Consequently, examining the work and home environment will probably be more relevant to reduce total sedentary time than investigating the neighbourhood environment. This has also been argued by the recently developed Systems of Sedentary time (SOS) framework of Chastin et al. [7]. In this framework, research priorities are identified, showing that examining the influence of institutional settings would be the main research focus at present [7].

Besides directly influencing transport-related and leisure-time sedentary behaviour, the neighbourhood might also be indirectly linked to sedentary behaviour. For example, it could have been that attractive neighbourhoods stimulate people to spend time outside, and in this way, reduce people’s sitting time at home (e.g. time spend watching television, or using a computer). However, as no associations were found, this hypothesis could not be confirmed.

Furthermore, it should be noted that the included physical environmental neighbourhood factors of the current study were predominantly micro-scale factors related to the neighbourhood streetscape. It might be that macro-scale factors, related to the neighbourhood design, do influence sedentary time in adults as suggested by Van Dyck et al. [9]. However, more research is needed.

The main strengths of this study include the use of the S-VAT to objectively measure physical environmental neighbourhood factors, as well as the use of accelerometers to objectively measure total sedentary time. Using objective measures has the advantage of being unaffected by recall or social desirability bias. The GT3X accelerometers, used in this study, have also an important limitation as they are not able to distinguish between postures, such as sitting and lying or standing still. Nevertheless, Koster et al. showed a high correlation for sedentary time between the GT3X accelerometer and the ActivPAL activity monitor, suggesting that time spent standing is limited [20]. Other limitations of the current study are its cross-sectional design, which does not allow for the establishment of causalities. Secondly, the relatively small study sample recruited from 24 neighbourhoods and consisting of relatively older and urban participants may have limited the power to detect meaningful associations and may limit the generalisability of our results. And finally, the lack of detailed information on objectively measured domain-specific sedentary times hindered us from drawing conclusions on correlates of domain-specific sedentary times. By using global positioning systems or wearable cameras next to accelerometers, determinants of domain-specific sedentary time could be determined objectively in future research [8].

## Conclusion

Our findings do not support associations of objectively measured physical environmental neighbourhood factors with adults’ objectively sedentary time in Dutch and Belgian adults. This is an important finding in itself and can inform future studies. Since most sedentary time occurs indoors, more research into the indoor environment at home and at work is recommended.
